# Prevalence and Patterns of Skin Diseases among School Children in Egypt: A National Cross-sectional Study

**DOI:** 10.1007/s44197-025-00440-8

**Published:** 2025-07-07

**Authors:** Azza Gaber Antar Farag, Zeinab Abdelaziz Kasemy, Ahmed Elsayed Elnemr, Areej Abdel Basset Hashish, Alzahraa Elsayed Mohamed, Reem Zahid Mohamed, Marian Adel Youssef Hanna, Monica Stef Said, Sara Gamal Badra, Seham Senosy Bar, Mai Medhat Mohamed Ghanem

**Affiliations:** 1 Dermatology, Andrology and STDs Department, Faculty of Medicine, Menoufia University, Shebin El-Kom, Menoufia, Egypt; 2 Public Health and Community Medicine Department, Faculty of Medicine, Menoufia University, Shebin El-Kom, Menoufia, Egypt; 3 Faculty of Medicine, Menoufia National University, Tukh tanbisha, Menoufia, Egypt; 4 Dermatology and Leprosy Clinic, Ministry of Health, Shebin El-Kom, Menoufia, Egypt; 5 Benha Hospital for Mental Health, Ministry of Health, Qalyubia, Egypt; 6 Birket Elsabe general hospital, Ministry of Health, Menoufia, Egypt; 7 Elshorok Medical Area, Ministry of Health, Cairo, Egypt; 8 Al Hadary Medical Center, Ministry of Health, Rod Al-Farag, Cairo, Egypt; 9 Shoubra Public Hospital, Ministry of Health, Cairo, Egypt; 10 Mansoura Health Insurance Organization, Ministry of Health, Mansoura, Egypt; 11 Dermatology Department, May 15 Hospital, Ministry of Health, Cairo, Egypt; 12 Faculty of Applied Health Sciences Technology, Menoufia National University, Tukh Tanbisha, Menoufia, Egypt

**Keywords:** Acne, Dermatoses, Pityriasis Alba, Pityriasis Versicolor, Prevalence, Skin Diseases, Wart

## Abstract

**Background:**

Pediatric dermatoses are common and impact the quality of life. This study aimed to estimate the prevalence and characteristics of pediatric dermatoses among 1^ry^ and 2^ry^ school students in Egypt.

**Methods:**

A Cross-sectional study was conducted on 23,203 1^ry^ and 2^ry^ school students of both sexes in eight governorates in Egypt between January 2023 and May 2023. A clinical examination for skin diseases was done, and a self-administered, author-designed questionnaire was given to children to complete with the assistance of their parents.

**Results:**

The average age of students was 12.00 ± 3.33 years. Prevalence of skin diseases was 37.9%. Positive family history of skin disease (aOR 3.482, 95% CI: 2.871–4.222, *p* < 0.001), sanitary water disposal (aOR 3.846, 95% CI: 3.001–4.930, *p* < 0.001), residence (aOR 1.760, 95% CI: 1.657–1.870, *p* < 0.001), father’s occupation and education (aOR 1.494, 95% CI: 1.334–1.672, *p* < 0.001 and aOR 1.349, 95% CI: 1.251–1.454, *p* < 0.001, respectively), and crowding index (aOR 1.469, 95% CI: 1.372–1.573, *p* < 0.001) were independent risk factors for exhibiting skin diseases.

**Conclusion:**

A high prevalence of pediatric dermatosis was established with associated sociodemographic risk factors, so healthcare and education programs and services should be directed toward children with continuous supervision and periodic examination.

**Supplementary Information:**

The online version contains supplementary material available at 10.1007/s44197-025-00440-8.

## Impact

Skin diseases are a significant public health issue among Egyptian schoolchildren, with a prevalence of 37.9% among 23,203 students.

This large-scale, multicenter study provides updated, nationally representative data on pediatric dermatoses in Egypt, identifying key sociodemographic and environmental risk factors previously underreported at the national level.

The findings support nationwide implementation of school-based dermatological screening and hygiene education programs to reduce disease burden and improve children’s health outcomes.

**Supplementary Information:**

The online version contains supplementary material available at 10.1007/s44197-025-00440-8.

## Introduction

The prevalence of pediatric dermatoses varies among communities depending on multiple factors, such as genetic predisposition, social class, parents’ employment and education, hygienic practices, health consciousness, and standard of medical treatment. These variables explain the different records in various communities and provide each group with a distinct pattern of illness. Researching pediatric dermatosis prevalence aims to evaluate health literacy and service accessibility to develop child health care plans that address real community needs [1].

Skin diseases are a significant source of health problems, affecting a high proportion of the population and causing distress and disabilities. Although very common in many developing countries, skin diseases are not often significant health problems, even when serious illnesses may be heralded by skin alterations [2].

Children at school frequently have skin conditions. Nonetheless, there is relatively little epidemiologic research on the broader public. The following are some of the drawbacks of several earlier studies: The validity of the diagnosis is dubious in self-administered questionnaire studies or surveys performed by nondermatologists; a survey of the referred cases in hospital-based settings may not fully reflect the actual situation in the general community [3].

Even though they are not life-threatening, skin conditions like alopecia areata and acne can be very upsetting for students in school. Families impacted by some chronic refractory skin disorders, for example, vitiligo, psoriasis, and atopic dermatitis may have major concerns about the psychological effects, course, and consequences of the illness and the therapeutic choices about long-term safety [4].

The prevalence of skin disorders has been studied through several hospital and community-based investigations, with the sorts of problems varying according to geography, climate, and participant age. Nonetheless, there is a few population-based research on prevalence of skin diseases conducted in Egypt. However, population-based investigations are necessary to understand the disease implications and direct suitable medical care toward them. In contrast, data from hospital-based research is restricted to individuals who see the hospital’s doctors with serious skin conditions. However, the kind and severity of skin disorders and illnesses varied significantly across research conducted in communities and schools [5].

This investigation aimed to determine the most common skin disorders among primary and secondary school students in Egypt, evaluate the sociodemographic determinants of their development, and raise awareness of the risk factors correlated with these conditions.

## Methods

### Study Design and Participants

A cross-sectional analytical investigation was carried out from January 2023 to May 2023, using a multistage random sampling procedure. Students aged 5 to 18 were recruited from 58 schools and selected randomly as part of the school health screening study. There was a balanced representation of all socioeconomic groups, considering the distribution of people who reside in rural and urban areas. The investigation’s sample was selected using data from the Egypt Demographic Health Survey conducted in 2015 [6].

### Sampling

**Fig. 1:**
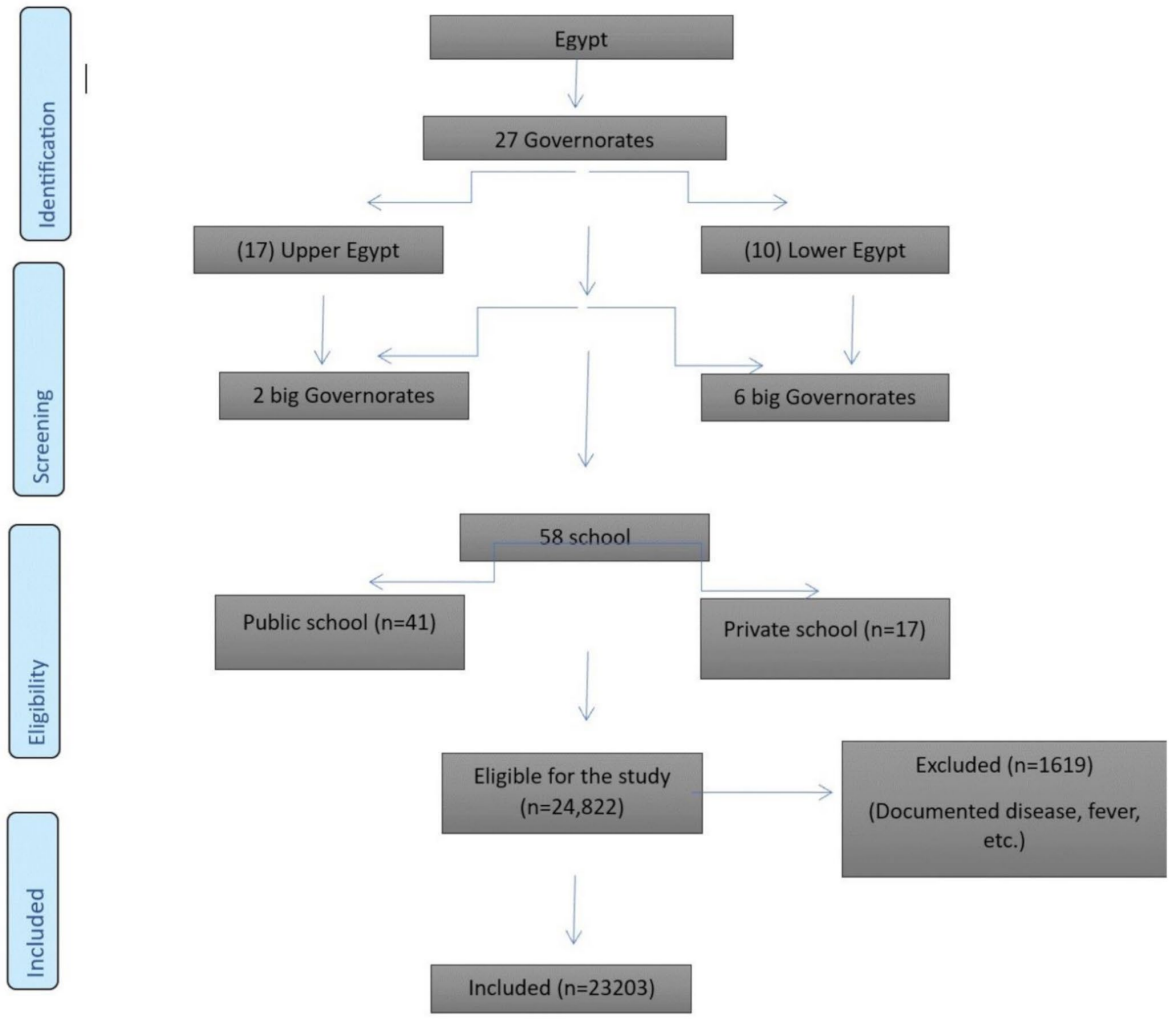
Flowchart for the sampling frame

Egypt is a nation comprised of 27 governorates, making it an impressive country. The investigation was conducted using a multistage random sampling technique. Initially, a selection was made among the 27 governorates, with two major governorates from Upper Egypt and six central governorates from Lower Egypt selected. Subsequently, a selection was made of 20 districts and cities, picked randomly from the previously selected governorates. The study conducted a full investigation of both private and public elementary and secondary schools within the districts and cities. A total of 58 facilities were randomly selected from the eight governorates included in the study (Fig. 1). The investigation was performed three days a week. The days with the highest population density were selected to expedite and gather a large amount of data. During both educational courses and break time, students were randomly examined in a school selected from a list of schools. We met the participants in the morning and in private rooms immediately after starting the education day. Author-designed questionnaire was given to children to be completed with the assistance of their parents for sociodemographic data **(Supplementary 1)**.

Between January 2023 and May 2023; 24,822 Egyptian students aged 5 to 18 were researched. To begin, we picked 23,423 adolescents and children who were eligible for the research, followed by 23,203 adolescents and children as a final total sample, excluding 220 children.

### Inclusion and Exclusion Criteria

Participants eligible for inclusion in the study were children aged between 6 and 18 years who were enrolled in a formal educational institution and attended classes at least three days per week. Only those whose parents or legal guardians provided written informed consent were included. Additionally, participants were required to be able to complete the survey and undergo the physical examination without experiencing undue psychological distress or physical discomfort. Children were excluded from the study if their parents or legal guardians declined to provide informed consent.

### Data Collection

All cases were exposed to the following.

A. Full history taking, which included: (i) personal history: name, sex, age, and residence, school type, stage of education, frequency of bathing per week, family size, parents’ occupation, and crowding index; (ii) present history: course, onset, relation to diet, period of dermatoses, relation to sun exposure, relation to stress, previous treatment, and history of other skin illnesses; (iii) family history of any skin illnesses.
B. Clinical examination, which included general examinations, bodyweight and height measurements, and a detailed dermatological examination of the entire body, including hair, nails, and mucous membranes. A clinical evaluation of skin illness was done to identify the distribution, clinical variants, and extent of the lesions. Wood’s light and dermoscopic examinations were done in case of doubtful diagnosis.

### Statistical Analysis

SPSS (Statistical Package for the Social Sciences) version 26 was used to tabulate and analyze the data collected on an IBM-compatible computer. Quantitative normally distributed data were represented as mean and SD and range, while qualitative information was expressed as number and percentage (No. and %). The relationship between two qualitative variables was investigated using the Pearson Chi-squared test (χ2) while the relationship between two quantitative normally distributed variables was examined using the student’s *t*-test (t). Kolmogorov-Smirnov Test was used to test the normality of quantitative data. To identify the predictors of skin illnesses among the participants in the investigation, logistic regression was used. Statistical significance was established at a *P*-value of less than 0.05.

## Results

This research involved 23,203 students aged 6 to 18 years with a mean of 12.00 ± 3.33, 11,237 males (48.4%) and 11,966 females (51.6%). A total of 72.2% were at public school, 79.5% were at primary stage, and 61.6% of the students were of urban residence. A total of 78% of students had a bath ≤ 2 per week, and 72.2% had hair wash ≤ 2 per week. The family history of skin diseases was positive in 2.4%. Regarding the students’ mothers, 51.7% of them were not working, and 51.7% got a university education (Table 1).

**Table 1:** Sociodemographic and lifestyle characteristics of studied participants (*n* = 23,203)

Variable	Mean ±SD	Range
Age (years)	12.00 ± 3.33	18-Jun
Family size	4.97 ± 1.11	11-Feb
House rooms	2.82 ± 0.68	6-Jan
Crowding index	1.84 ± 0.52	0.5-6
Bathing per week	1.83 ± 0.76	3-Jan
Hair wash per day	1.96 ± 0.77	3-Jan
BMI (kg/m^2^)	20.55 ± 3.85	9.72–39.84
	**No.**	**%**
Sex		
Male	11237	48.4
Female	11966	51.6
School type		
Public	16747	72.2
Private	6456	27.8
Stage of education		
Primary	18451	79.5
Secondary	4752	20.5
Residence		
Urban	14291	61.6
Rural	8912	38.4
Frequency of bathing per week		
≤ 2	18104	78
> 2	5099	22
Frequency of hair wash per week		
≤ 2	16871	72.7
> 2	6332	27.3
Positive family history	568	2.4
Mother occupation		
Working	11209	48.3
Not working	11994	51.7
Mother’s education		
Illiterate	1406	6.1
Basic	2118	9.1
Secondary	7024	30.3
University	11997	51.7
Postgraduate	658	2.8
Father occupation		
Working	21026	90.6
Not working	2177	9.4
Father’s education		
Illiterate	997	4.3
Basic	1968	8.5
Secondary	5116	22
University	14140	60.9
Postgraduate	982	4.2
Water disposal	20311	87.5
Waste disposal	20909	90.1

SD: standard deviation; BMI: body mass index

**Fig. 2:**
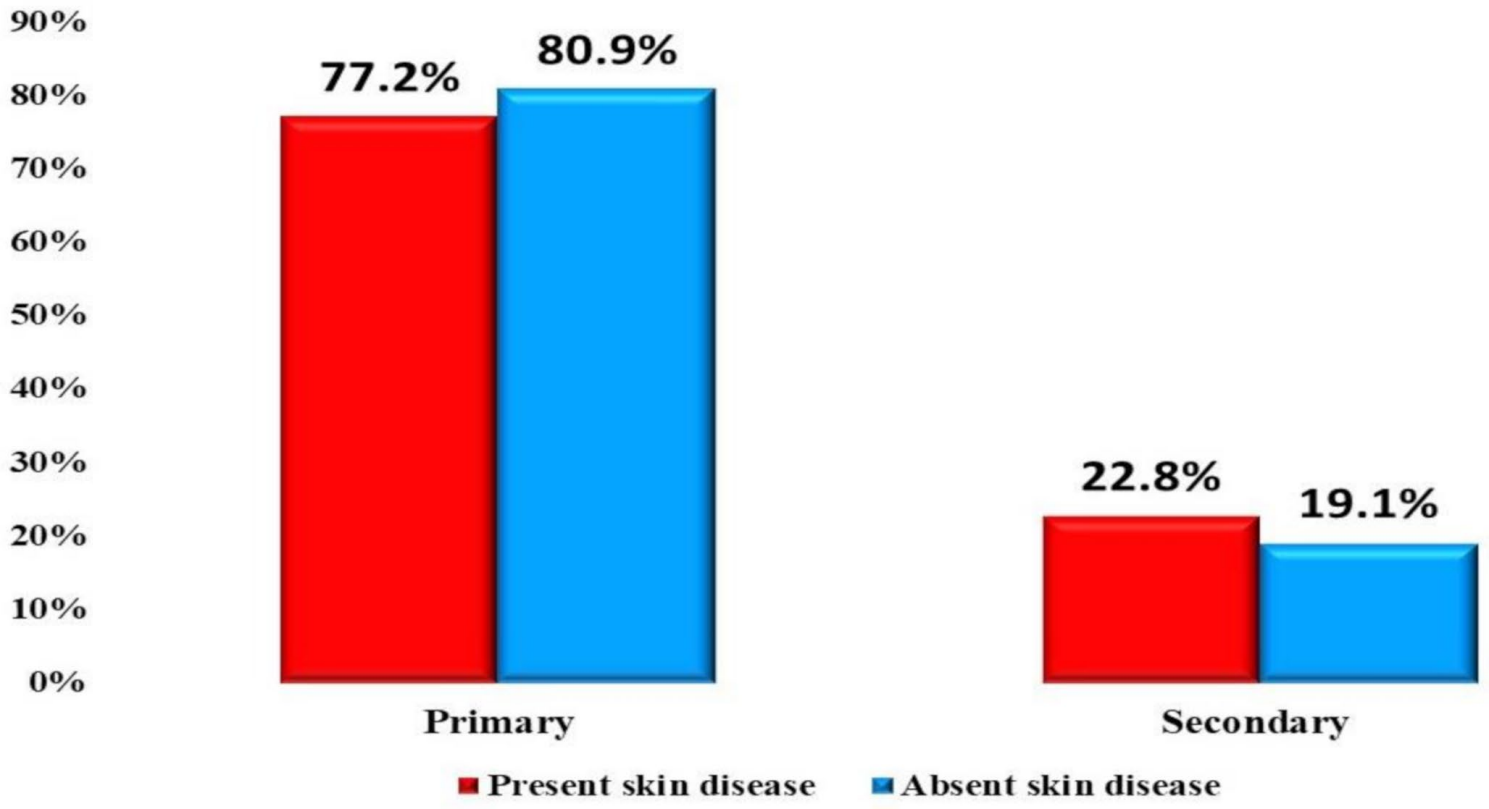
Distribution of skin diseases according to the education stage

Skin diseases were observed in 8787 students (37.9%). A statistically significant difference was noticed between those with and without skin disease as skin diseases, which were higher in students with a mean age of 12.34 ± 3.40 years; those in public schools (74.1%); those in primary stage (77.2%) (Fig. 2); those who live in urban residence (53.8%); those having less frequency of bathing per week (76.3%); those with a positive family history of skin diseases (4.7%); those not having working mothers (57.5%); those having working fathers (93.3%); those having a mother (46.3%) and a father (55.2%) with university education; those with a family size of mean of 5.01 **±** 1.18; those with fewer house rooms; and those with more crowding index and BMI (Table 2).

**Table 2:** Distribution of skin disease regarding sociodemographic data (*n* = 23,203)

Characteristic	Skin disease occurrence				Test of sign	
	Present (*n* = 8,787)		Absent (*n* = 14,416)			
	No.	%	No.	%	χ^2^	*p*-value
Sex					1.52	0.218
Male	4301	48.9	6936	48.1		
Female	4486	51.1	7480	51.9		
School type					26.33	< 0.001*
Public	6512	74.1	10235	71		
Private	2275	25.9	4181	29		
Stage of education					47.56	< 0.001*
Primary	6782	77.2	11669	80.9		
Secondary	2005	22.8	2747	19.1		
Residence					362.29	< 0.001*
Urban	4728	53.8	9563	66.3		
Rural	4059	46.2	4853	33.7		
Frequency of bathing/week					23.4	< 0.001*
≤ 2	6708	76.3	11396	79.1		
> 2	2079	23.7	3020	20.9		
Frequency of hair wash/week					2.99	0.084
≤ 2	6446	73.4	10425	72.3		
> 2	2341	26.6	3991	27.7		
Positive family history	415	4.7	153	1.1	306.5	< 0.001*
Mother occupation					189.19	< 0.001*
Working	3737	42.5	7472	51.8		
Not working	5050	57.5	6944	48.2		
Mother’s education					329.72	< 0.001*
Illiterate	740	8.4	666	4.6		
Basic	942	10.7	1176	8.2		
Secondary	2674	30.4	4350	30.2		
University	4070	46.3	7927	55		
Postgraduate	361	4.1	297	2.1		
Father occupation					116.39	< 0.001*
Working	8195	93.3	12831	89		
Not working	592	6.7	1585	11		
Father’s education					466.4	< 0.001*
Illiterate	620	7.1	377	2.6		
Basic	805	9.2	1163	8.1		
Secondary	1965	22.4	3151	21.9		
University	4851	55.2	9289	64.4		
Postgraduate	546	6.2	436	3		
Water disposal, yes	8264	94	12047	83.6	549.69	< 0.001*
Waste disposal, yes	8342	94.9	12567	87.2	369.16	< 0.001*
	**Mean ± SD**		**Mean ± SD**		**Test of sig.**	***p*** **-value**
Age (years)	12.34 ± 3.40		11.80 ± 3.26		t = 12.19	< 0.001*
Family size	5.01 ± 1.18		4.95 ± 1.06		t = 4.12	< 0.001*
House rooms	2.70 ± 0.67		2.89 ± 0.67		t = 21.30	< 0.001*
Crowding index	1.95 ± 0.58		1.78 ± 0.47		t = 23.79	< 0.001*
BMI (kg/m^2^)	20.87 ± 4.15		20.36 ± 3.64		t = 10.09	< 0.001*

*: statistically significant, χ^2^: chi-squared test, t: Student’s *t*-test, SD: standard deviation, BMI: body mass index

The most frequent skin illnesses were acne vulgaris, with a percentage of 5.6%, followed by pityriasis alba (4.3%), wart (4.2%), pityriasis versicolor (3.7%), and papular urticaria (3.5%) (Table 3).

**Table 3:** Distribution of skin diseases among the studied participants (*n* = 23,203)

Skin disease		No.	%
All		**8787**	**37.9**
Infectious dermatosis		4299	18.5
Viral	Wart	976	4.2
	Herpes simplex	652	2.8
	Chicken pox	84	0.4
	Molluscum contagiosum	18	0.1
Fungal	Pityriasis versicolor	867	3.7
	White piedra	82	0.4
Parasitic	Pediculosis	490	2.1
	Scabies	470	2
Bacterial	Folliculitis	365	1.6
	Impetigo	295	1.3
Non infectious		8870	
Pilosebaceous	Acne	1306	5.6
	Alopecia areata	660	2.8
	Traction alopecia	423	1.8
	Dandruff	241	1
	Telogen effluvium	116	0.5
Dermatitis	Pityriasis alba	1008	4.3
	Papular urticaria	804	3.5
	Seborrheic dermatitis	647	2.8
	Eczema	645	2.8
	Angular stomatitis	633	2.7
	Atopic dermatitis	633	2.7
	Contact dermatitis	265	1.1
	Berloque dermatitis	52	0.2
Papulosquamous	Psoriasis	171	0.7
	Lichen planus	128	0.6
Pigmentary disorders	Vitiligo	209	0.9
	Nevi	133	0.6
	Freckles	102	0.4
Genodermatosis	Neurofibromatosis and others	113	0.5
Miscellaneous	Hemangioma	148	0.6
	Acanthosis nigricans	137	0.6
	Nail dystrophy	108	0.5
	Milia	106	0.5
	Chilblain	34	0.1
	Lipoma	25	0.1
	Keloid	23	0.1

Multiplicities of dermatoses per child should be taken into consideration to avoid false perceptions of some discrepancies in frequencies and percentages

The prevalence of acne vulgaris was statistically significantly greater in women than that in men. In comparison, the prevalence of pityriasis alba, wart, and tinea versicolor was statistically significantly greater in men than that in women (*p*-value < 0.05) (Table 4).

**Table 4:** The most common skin disease in relation to sex among studied participants (*n* = 23,203)

Skin disease	Sex			
	Male (*n* = 11,237)		Female (*n* = 11,966)	
	No.	%	No.	%
Acne vulgaris	529	4.7	777	6.5
Pityriasis alba	659	5.9	349	2.9
Wart	536	4.8	440	3.7
Tinea versicolor	456	4.1	411	3.4
Papular urticaria	385	3.4	419	3.5

**Fig. 3:**
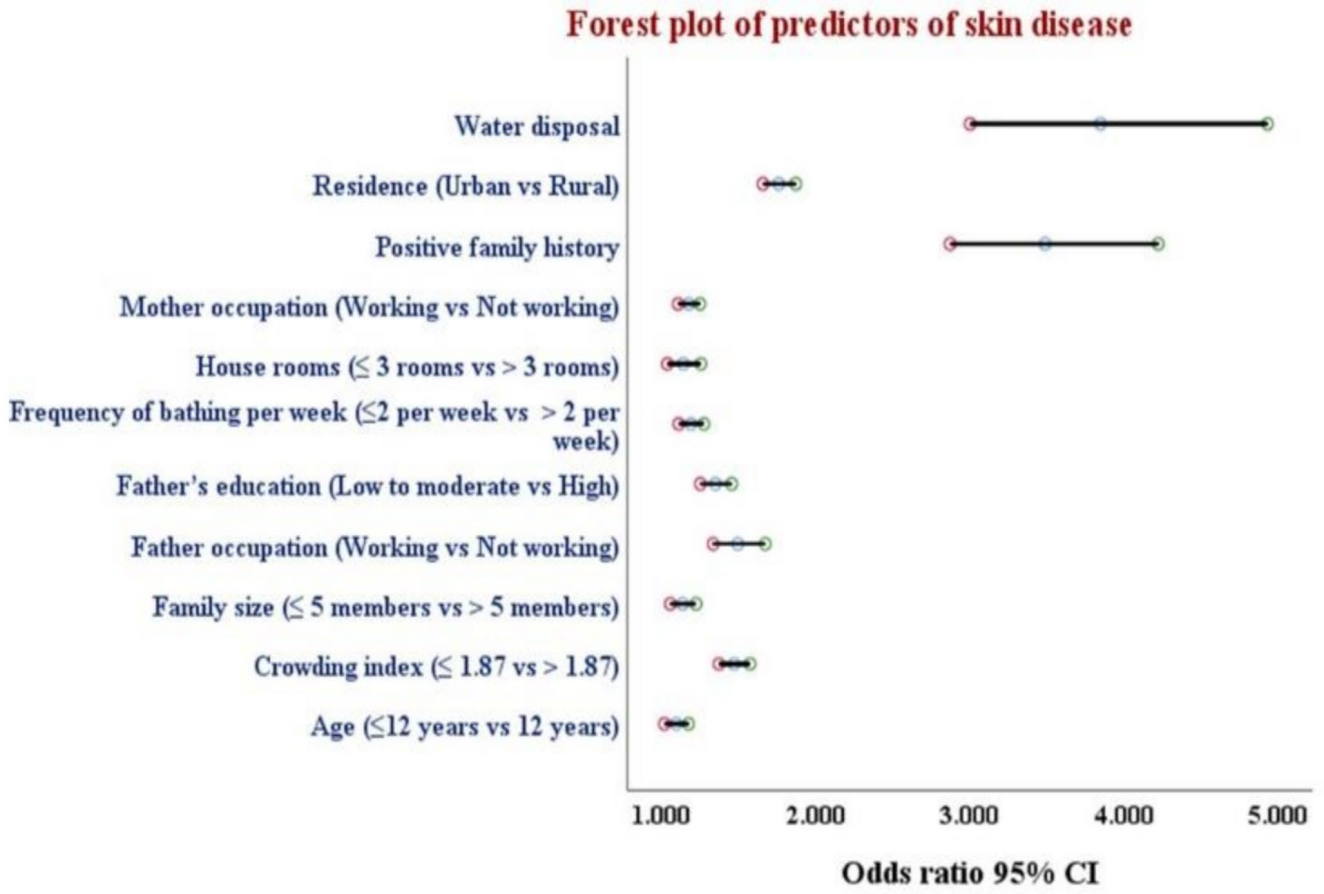
Forest plot for crude odds ratios and their 95% CIs

Binary logistic regression analysis was performed to confirm the effects of school type, educational stage, residence, frequency of bathing per week, positive family history of skin disease, mother occupation and education, father occupation and education, water and waste disposal, age, family size, number of house rooms, and crowding index and BMI on the likelihood that the student will exhibit a skin disease. As per univariable analysis, all predictor variables were statistically significant. Accordingly, all statistically significant predictor variables on univariable analysis were entered into a regression model. Residence (aOR 1.760, 95% CI: 1.657–1.870, *p* < 0.001), frequency of bathing per week (aOR 1.190, 95% CI: 1.111–1.274, *p* < 0.001), positive family history of skin disease (aOR 3.482, 95% CI: 2.871–4.222, *p* < 0.001), mother’s occupation (aOR 1.176, 95% CI: 1.107–1.249, *p* < 0.001), father’s occupation and education (aOR 1.494, 95% CI: 1.334–1.672, *p* < 0.001 and aOR 1.349, 95% CI: 1.251–1.454, *p* < 0.001, respectively), water disposal (aOR 3.846, 95% CI: 3.001–4.930, *p* < 0.001), age (aOR 1.093, 95% CI: 1.018–1.174, *p* = 0.014), family size (aOR 1.136, 95% CI: 1.056–1.223, *p* = 0.001), number of house rooms (aOR 1.140, 95% CI: 1.037–1.254, *p* = 0.007), and crowding index (aOR 1.469, 95% CI: 1.372–1.573, *p* < 0.001) were independent risk factors for exhibiting skin diseases (Table 5; Fig. 3).

**Table 5:** Predictors of the likelihood of occurrence of skin disease

Predictor	Univariable			Multivariable		
	cOR	95% CI	*p*-value	aOR	95% CI	*p*-value
School type (private)	1.169	1.101–1.241	< 0.001*	1.001	0.937–1.069	0.983
Stage of education (primary)	1.256	1.177–1.340	< 0.001*	1.037	0.957–1.124	0.378
Residence (urban)	1.692	1.602–1.786	< 0.001*	1.760	1.657–1.870	< 0.001*
Frequency of bathing/week (≤ 2)	1.170	1.098–1.246	< 0.001*	1.190	1.111–1.274	< 0.001*
Positive family history (no)	4.621	3.832–5.573	< 0.001*	3.482	2.871–4.222	< 0.001*
Mother occupation (working)	1.454	1.378–1.534	< 0.001*	1.176	1.107–1.249	< 0.001*
Mother’s education (high)	1.306	1.238–1.377	< 0.001*	1.002	0.932–1.077	0.959
Father occupation (not working)	1.710	1.550–1.887	< 0.001*	1.494	1.334–1.672	< 0.001*
Father’s education (high)	1.302	1.232–1.376	< 0.001*	1.349	1.251–1.454	< 0.001*
Water disposal (no)	3.107	2.815–3.430	< 0.001*	3.846	3.001–4.930	< 0.001*
Waste disposal, yes	2.758	2.478–3.070	< 0.001*	0.827	0.629–1.086	0.172
Age (≤ 12 years)	1.316	1.248–1.388	< 0.001*	1.093	1.018–1.174	0.014*
Family size (≤ 5 members)	1.378	1.298–1.462	< 0.001*	1.136	1.056–1.223	0.001*
House rooms (> 3 rooms)	1.646	1.520–1.783	< 0.001*	1.140	1.037–1.254	0.007*
Crowding index (≤ 1.87)	1.903	1.804–2.008	< 0.001*	1.469	1.372–1.573	< 0.001*
BMI (kg/m^2^) (≤ 19.82)	1.216	1.153–1.283	< 0.001*	1.048	0.983–1.117	0.150

cOR = crude odds ratio. aOR = adjusted odds ratio. CI = confidence interval. () = reference category

## Discussion

Pediatric dermatoses are common among school children, especially in developing countries. It can cause psychological distress, loss of self-confidence, and school abstinence, especially with affection of apparent parts of the skin [7].

In Egypt, few school-based investigations have been done by dermatologists to identify the prevalence of skin diseases, and all investigations were conducted in small numbers and in only one governorate [7–9]. The current study was conducted on a relatively larger number of children in eight governorates.

This study involved students aged 6 to 18 years, including 11,237 male and 11,966 female students. A skin condition was detected in 8,787 students, accounting for 37.9% of the total population. This finding aligns with earlier research conducted in African countries, where the prevalence of skin diseases ranged from 39.6 to 40.2% [10, 11]. However, subsequent investigations demonstrated a greater incidence of skin problems, precisely 76% and 60%, respectively [7, 12]. Other studies revealed a lower prevalence of skin diseases, 23.8% and 26.7% [8, 13]. These variabilities may be due to different climatic, social, and hygienic factors.

In this study, the prevalence of skin illnesses was significantly higher in primary school students, as in the study conducted in Turkey [14]. This may be related to the large number of children included in the research who were at the primary stage, as primary education in Egypt is obligatory and includes a larger number of children than the secondary stage, which is more representative.

In a previous study in Iraq, 53.8% of students had skin diseases in urban areas similar to this study [15]. However, other studies demonstrated a higher prevalence in rural areas due to lower socioeconomic standards and poor sanitation [7, 9].

In this study, we demonstrated a statistically significant variation between those with and without skin disease regarding the type of school, as it was higher in public schools. In line with this, **Ewurum et al.** discovered a greater prevalence of skin illnesses documented among children in public schools compared to private schools [11]. Moreover, **Mohammed and colleagues** in Sudan documented a prevalence of 45.5 and 25% for public and private schools, respectively [16]. Similarly, **Laczynski and Cestari** in Brazil documented prevalence figures of 93% and 83%, respectively, for public and private schools [17]. In this study, most public-school pupils were of low socioeconomic class, which may explain why skin diseases were more common among them. Risk factors for skin illnesses, such as poor hygiene and overcrowding, have been associated with low socioeconomic status.

In this study, noninfectious dermatoses were 38.2%, like reports in Iraq 33.7% [18] and Tanzania 35.4% [19], and infectious dermatoses were 18.9%, like the report in Jaipur 19.8% [12]. Infectious dermatoses may be underestimated in cross-sectional studies due to seasonal variation in disease occurrence, home isolation of diagnosed cases, and home care in febrile conditions. Moreover, the lack of seeking medical advice for most noninfectious dermatoses, as lesions are asymptomatic or due to ignorance, led to an increase in their prevalence.

The most common skin illnesses observed in this research were acne vulgaris, with a percentage of 5.6%, followed by pityriasis alba 4.3%, wart 4.2%, pityriasis versicolor 3.7%, and papular urticaria 3.5%.

The most common skin disease recorded in this study was acne at 5.6%, which was significantly higher in females; these discoveries were in line with research conducted in Turkey that revealed an acne prevalence of 6% and more in females [14]. Furthermore, another study in Hong Kong showed that acne was the most frequent skin illness among school kids 9.8% [20]. This could be explained by including students in the adolescent period up to 18 years. In other investigations, the prevalence figures were 2.7% in Brazil and Ethiopia [17, 21]. This distinction can be explained by the racial variances, genetic susceptibility, hormonal profile, and dietary habits that play a significant role in acne vulgaris occurrence.

Acne was present in students’ age ranging from 15 to 18 and represents 22% of students of the same age. Another study in secondary school in Egypt revealed a prevalence of clinically confirmed acne of 24.4%, with greater rates among women that may be due to hormonal changes throughout menstruation or greater levels of stress among women [22]. Acne is a multifaceted illness, and the study only assessed acne cases for three months during the winter (January to May). Notably, the production of skin surface lipids declines in the winter, and there is a negative association between lipids and acne incidence. Patients with acne were found to have psychological, social, and emotional problems that were as marked as those described by cases with chronic disabling asthma, diabetes, epilepsy, arthritis, and back pain. Similar community studies in secondary school children reported a higher level of psychiatric problems [23, 24].

The second most frequent illness was pityriasis alba, with a prevalence of 4.3%, which was significantly higher in males. In a previous study in Ismailia City, Egypt, 10.3% of primary school students had pityriasis alba [25]. This may be due to skin dryness due to sun exposure, going to school on foot, and being more prevalent in males as they are more exposed to sunlight during outdoor play and helping parents at work.

The prevalence of warts in this research was 4.2%, similar to the prevalence of 4.6% reported by **Mengist Dessie et al.** in Northern Ethiopia [26]. This may be due to sharing shoes and clothes and barefoot activities. Lower prevalence of 3.3% and 2.6% were documented in Saudi Arabia [27] and Turkey, respectively [14].

The prevalence of pityriasis versicolor in this research was 3.7%, comparable with that reported by **Mengist Dessie et al.** [26] in Northern Ethiopia at 6.3% and by **Fung and Lo** [20] in Hong Kong at 4.5%. Pityriasis versicolor may be explained by the inclusion of older students up to 18 years of age in this study. The stimulation of sex hormones, such as androgen stimulation, that occurs in adolescence results in greater development of the sebaceous gland with more secretion of sebum and skin lipids, which favors the growth of *Malassezia*, the etiologic agent of pityriasis versicolor. Moreover, the moist and humid environment is commonly encountered throughout physical activity by the adolescent group [28].

Noninfective skin lesions, such as papular urticaria, were in the fifth rank, with a prevalence of 3.5%, similar to that reported in Jaipur [12]. Papular urticaria results from an exaggerated response to insect bites and stings. It may be worsened by poor environmental sanitation and inadequate drainage prevalent in developing countries like Egypt [29].

In this study, skin diseases were significantly associated with risk factors, such as frequency of bathing per week, family size, house rooms, and crowding index like the study by **Alkalash et al.** [7]. Additionally, **Ewurum et al.** discovered that using a multivariate logistic regression and identified the number of individuals per room, the frequency of bathing per week, and the source of water as important risk variables for skin illnesses [11]. The study conducted by **Dessie et al.** found a strong association between skin illnesses and factors such as history of skin illnesses and personal hygiene [26].

Skin diseases were significantly associated with parents’ occupations. In line with our study findings, in Ethiopia, **Lulu et al.** [21] and **Alkalash et al.** [7] also discovered a significant correlation between skin illness and parents’ occupations.

### Study Strengths and Limitations

This research was performed on a large sample size covering the entire country. However, this study had a one-point inspection of the skin for illnesses, which may have overlooked the students with infectious skin disorders for a short period, underestimating the real burden of skin illnesses in these kids.

## Conclusion

Skin disorders were prevalent among schoolchildren. Living conditions and sociodemographic factors are significant risk factors for skin illnesses. The high prevalence of skin illnesses in our environment can be reduced by implementing standard hygienic practices by school students and their parents/caregivers. Consequently, we advocated for implementing a preventive health education program that would educate school students at various levels, as well as their families and teachers, regarding skin diseases.

## Electronic Supplementary Material

Below is the link to the electronic supplementary material.

Supplementary Material 1

## Data Availability

No datasets were generated or analysed during the current study.

## References

[1] Balai M, Khare AK, Gupta LK, Mittal A, Kuldeep CM. Pattern of pediatric dermatoses in a tertiary care centre of South West Rajasthan. Indian J Dermatol. 2012;57:275–8. 10.4103/0019-5154.97665 22837560 10.4103/0019-5154.97665 PMC3401841

[2] Cortés H, Rojas-Márquez M, Del Prado‐Audelo ML, Reyes‐Hernández OD, González‐Del Carmen M, Leyva‐Gómez G. Alterations in mental health and quality of life in patients with skin disorders: a narrative review. Int J Dermatol. 2022;61:783–91. 10.1111/ijd.15852 34403497 10.1111/ijd.15852

[3] Paller AS, Mancini AJ. Paller and Mancini-Hurwitz clinical pediatric dermatology: A textbook of skin disorders of childhood and adolescence. 6th ed. Elsevier Health Sciences; 2020.

[4] Flohr C, Hay R. Putting the burden of skin diseases on the global map. Br J Dermatol. 2021;184:189–90. 10.1111/bjd.19704 33544440 10.1111/bjd.19704

[5] Davila M, Christenson LJ, Sontheimer RD. Epidemiology and outcomes of dermatology in-patient consultations in a Midwestern US university hospital. Dermatol Online J. 2010;16:12. 10.5070/D364h8j3kz 20178708

[6] El-Zanaty F, Way A. Egypt health issue survey. Ministry of Health and Population, Cairo, Egypt. 2015. https://dhsprogram.com/pubs/pdf/FR313/FR313.pdf

[7] Alkalash SH, Gaber MA, Kamal AA. Prevalence of skin diseases among primary school children in Benha city, Kalubia governorate, Egypt. Menoufia Med J. 2023;36:21. 10.59204/2314-6788.1020

[8] El-Khateeb EA, Lotfi RA, Abdel‐Aziz KM, El‐Shiekh SE. Prevalences of skin diseases among primary schoolchildren in damietta, Egypt. Int J Dermatol. 2014;53:609–16. 10.1111/ijd.12335 24758232 10.1111/ijd.12335

[9] El-Dawela RE, Fatehy AN, Abd Elmoneim AA. Prevalence of skin diseases among school children: a survey in the Sohag Governorate. J Egypt Women Dermatol Soc. 2012;9:47–51. 10.1097/01.EWX.0000407242.66890.d4

[10] Amoran OE, Runsewe-Abiodun OO, Mautin AO, Amoran IO. Determinants of dermatological disorders among school children in sagamu, Nigeria. Educ Res. 2011;2:1743–8. http://www.interesjournals.org/ER

[11] Ewurum O, Ibeneme CA, Nnaji TO, Ikefuna AN. Spectrum of skin disorders among primary school children in umuahia, South-East Nigeria. Niger J Clin Pract. 2022;25:1076–82. 10.4103/njcp.njcp_1573_21 35859468 10.4103/njcp.njcp_1573_21

[12] Nijhawan M, Bagri M, Nijhawan S, Bishnoi S, Agarwal S, Nijhawan S. Pattern of common skin diseases among school going children in Semi-Urban area of jaipur: A cross-sectional study. Indian J Paediatr Dermatol. 2020;21:275–8. 10.4103/ijpd.IJPD_119_19

[13] Hogewoning A, Amoah A, Bavinck JN, Boakye D, Yazdanbakhsh M, Adegnika A, et al. Skin diseases among schoolchildren in ghana, gabon, and Rwanda. Int J Dermatol. 2013;52:589–600. 10.1111/j.1365-4632.2012.05822.x 23557028 10.1111/j.1365-4632.2012.05822.x

[14] Uludağ A, Kılıc SO, Isık S, Ertekin YH, Tekin M, Cevizci S, et al. Prevalence of skin disorders in primary and secondary school age children in canakkale, turkey: a community-based survey. Adv Dermatology Allergology/Postępy Dermatol Alergol. 2016;33:176–81. 10.5114/ada.2016.60610 10.5114/ada.2016.60610 PMC4969413 27512351

[15] Algharbawi BA, Baraznchi SJ, Al-Mnehil JK. Prevalence and pattern of skin disorder among primaryschool children in Wasit governorate, iraq, 2021. Tex J Med Sci. 2023;17:46–50. 10.62480/tjms.2023.vol17.pp46-50

[16] Mohammed SA, Elhassan MM, Hussein K. The pattern of paediatric dermatoses among primary school children in Khartoum north, 2007. Sudan J Public Health. 2010;5:182–6. https://www.cabidigitallibrary.org/doi/full/10.5555/20113151447

[17] Laczynski CM, Cestari SD. Prevalence of dermatosis in scholars in the region of ABC Paulista. Bras Dermatol. 2011;86:469–76. 10.1590/S0365-05962011000300008 10.1590/s0365-05962011000300008 21738963

[18] Khalifa KA, Al-Hadithi TS, Al-Lami FH, Al-Diwan JK. Prevalence of skin disorders among primary-school children in Baghdad governorate, Iraq. East Mediterr Health J. 2010;16:209–13. 10.26719/2010.16.2.209 20799576

[19] Amiri M, Furia FF, Bakari M. Skin disorders among children living in orphanage centres in Dar Es salaam, Tanzania. Trop Med Health. 2020;48:1–7. 10.1186/s41182-020-00216-9 32377156 10.1186/s41182-020-00216-9 PMC7191825

[20] Fung WK, Lo KK. Prevalence of skin disease among school children and adolescents in a student health service center in Hong Kong. Pediatr Dermatol. 2000;17:440–6. 10.1046/j.1525-1470.2000.01841.x 11123774 10.1046/j.1525-1470.2000.01841.x

[21] Lulu Y, Tolesa G, Cris J. Prevalence and associated factors of skin diseases among primary school children in illuababorzone, oromia regional state, South West Ethiopia. Indo Am J Pharm Res. 2017;7:7374–83. 10.1046/j.1525-1470.2000.01841.x

[22] Tayel K, Attia M, Agamia N, Fadl N. Acne vulgaris: prevalence, severity, and impact on quality of life and self-esteem among Egyptian adolescents. J Egypt Public Health Assoc. 2020;95:1–7. 10.1186/s42506-020-00056-9 33165744 10.1186/s42506-020-00056-9 PMC7642029

[23] Kilkenny M, Stathakis V, Hibbert ME, Patton G, Caust J, Bowes G. Acne in Victorian adolescents: associations with age, gender, puberty and psychiatric symptoms. J Paediatr Child Health. 1997;33:430–3. 10.1111/j.1440-1754.1997.tb01635.x 9401889 10.1111/j.1440-1754.1997.tb01635.x

[24] Smithard A, Glazebrook C, Williams HC. Acne prevalence, knowledge about acne and psychological morbidity in mid-adolescence: a community‐based study. Br J Dermatol. 2001;145:274–9. 10.1046/j.1365-2133.2001.04346.x 11531791 10.1046/j.1365-2133.2001.04346.x

[25] Elshafey WS, Fiala LA, Mohamed RW, Ismael NA. The distribution and determinants of pityriasis Alba among elementary school students in Ismailia City. J Am Sci. 2012;8:444–9. https://www.scribd.com/document/419479582/Psoriasis

[26] Mengist Dessie A, Fenta Feleke S, Getaye Workie S, Getinet Abebe T, Mossu Chanie Y, Kassa Yalew A. Prevalence of skin disease and its associated factors among primary schoolchildren: A Cross-Sectional study from a Northern Ethiopian town. Clin Cosmet Investig Dermatol. 2022;29:791–801. 10.2147/CCID.S361051 10.2147/CCID.S361051 PMC9063791 35521561

[27] Amin TT, Ali A, Kaliyadan F. Skin disorders among male primary school children in al hassa, Saudi arabia: prevalence and socio-demographic correlates-a comparison of urban and rural populations. Rural Remote Health. 2011;11:52–64. 10.3316/informit.338710170158012 21355670

[28] He SM, Du WD, Yang S, Zhou SM, Li W, Wang J, et al. The genetic epidemiology of Tinea versicolor in China. Mycoses. 2008;51:55–62. 10.1111/j.1439-0507.2007.01437.x 18076596 10.1111/j.1439-0507.2007.01437.x

[29] Bahamdan K, Mahfouz A, Tallab T, Badawi IA, Al-Amari OM. Skin diseases among adolescent male boys in abha, Saudi Arabia. Int J Dermatol. 1996;35:405–7. 10.1111/j.1365-4362.1996.tb03020.x 8737873 10.1111/j.1365-4362.1996.tb03020.x

