# Supplementary material for: Prevalence and Patterns of Skin Diseases among School Children in Egypt: A National Cross-sectional Study
Source: J Epidemiol Glob Health. 2025 Jul 7;15(1):94. doi: 10.1007/s44197-025-00440-8 (PMC12234915; doi:10.1007/s44197-025-00440-8)
Supplement: Supplementary file 1 — Supplementary Material 1 [file 44197_2025_440_MOESM1_ESM.docx]

**Supplementary 1: Prevalence of skin diseases among school children questionnaire**

***Sociodemographic data:***

1. **Name:**
2. **Age:**
3. **Sex: - Male - Female**
4. **Stage of education: - Primary -Secondary**
5. **School type: -Public -Private**
6. **Residence: - Urban - Rural**
7. **Mother’s education: -Illiterate - Basic - Secondary -University – Postgraduate**
8. **Father’s education: -Illiterate - Basic - Secondary -University - Postgraduate**
9. **Mother’s occupation: - Working - Not working**
10. **Father’s occupation: - Working - Not working**
11. **Income: - Not enough - Enough - More than Enough**
12. **Family size:**
13. **No of house rooms:**
14. **Crowding index: family size/ house rooms**
15. **Water disposal: Yes No**
16. **Waste disposal: Yes No**
17. **Bathing week: once/ week - twice/week - 3 times/ week**
18. **Hair washing: once/ week - twice/week - 3 times/ week**
19. **Family history of skin diseases: - yes - No. If yes: Which skin disease…………………………………………………………………………**
20. **BMI: Weight ……. Height: ……………….**
